# State of knowledge of Cameroonian drug prescribers on pharmacovigilance

**DOI:** 10.11604/pamj.2015.20.70.3873

**Published:** 2015-01-27

**Authors:** Francis Nde, Aimé Bernard Djitafo Fah, Francis Ampère Simo, Denis Wouessidjewe

**Affiliations:** 1 Acute Medicine, Medicine of Catastrophes, Public Health, Epidemiology, Biostatistics, Pharmaceutical Medicine, Faculty of Medicine and Pharmaceutical Sciences, at University of Douala, Douala, Cameroon; 2 UDM, Bangante, Cameroon; 3 University of Yaounde I, UdM, Bangante, Cameroon; 4 Joseph Fourier de Grenoble University, UFR de Pharmacology, Department Molecular Pharmacology, UMR 5063, Bât. E (André Rassat), Pôle chimie – BP53, 38041 Grenoble

**Keywords:** Pharmacovigilance system, knowledge, practices, Cameroon, drug side effect

## Abstract

**Introduction:**

The present study conducted in Cameroon from June 2013 to February 2014 aimed to estimating the level of pharmacovigilance knowledge and practice of health professionals in Cameroon.

**Methods:**

We conducted a descriptive cross-sectional survey on 149 health professionals in Cameroon from June to September 2013. Data were analyzed using software IBM SPSS 20.0. We calculated proportions and odd ratio, and confident interval of their values, keeping a threshold of p of 0.05 to determine the level of significance.

**Results:**

Ninety percent (90%) of declaration of side effects were made to the medical representatives and 4% to the National Pharmacovigilance Centre. Fifty four percent (54%) of physicians were not aware of the existence of a National Pharmacovigilance system. Ten (10%) of prescribers had never heard of pharmacovigilance, however respondents answered unanimously that they need training on pharmacovigilance. A wrong definition was given by most of the nurses and dentists (61,1% and 58,3% respectively) as compared to physicians and pharmacists (respectively 15.2% and 26,5%). Given the results of this study, the establishment of a National Pharmacovigilance system based on a solid legal foundation is necessary in Cameroon. This implementation must go through the involvement of all stakeholders and their awareness raising on the importance of this activity and its positive impact on the health of populations.

**Conclusion:**

Pharmacovigilance is a public health problem in Cameroon, with due to lack of good knowledge and practice of prescribers, precisely physicians, pharmacists, nurses, and dentists who are not always aware of an existing pharmacovigilance system in Cameroon.

## Introduction

Durgs side effect has been widely studied through pharmacovigilance studies since the thalidomide problem [1]. Pharmacovigilance is widely spread and applied in European countries [2, 3] and USA [4], and more and more in developing countries [5] where the health system makes a case of health professional training [6]. Drugs are widely used nowadays to fight diseases that threaten the lives and well- being of people in the world, particularly in Africa and hence the Cameroon. In this country, the drug represents a burden for patient as it could already be observed in a study since 1997 [7]. Though helpful, drugs have negative aspects, with possible reduction of patients of life of patients, hospital admissions and prolongation of hospital stays, and in extreme cases, may cause death. Although before a drug is put on the market it has to go through number of trials, there is no enough experience to watch all the possible side effects it may cause, especially in certain categories of populations like children and pregnant women, or in association with other drugs. Thus a need to follow up drugs side effect after it acceptance on the market, since the number of patients increases significantly and becomes practically unlimited. In this context, insignificant and undetectable in clinical trials adverse reactions may occur and should be seriously considered. However, besides monitoring the side effects of drugs like those developed by WHO through Cohort Event Monitoring, monitoring medication side effects is not sufficiently developed Sub-Saharan Africa [8]. Several pilot projects have been attempted in this part of Africa, showing in most of cases that pharmacovigilance is a major public health problem, and the need of improvement action to improve the situation [9]. Majority of these pilot projects are usually related to vertical programs malaria, ARV or vaccination types. In addition, they often reveal gaps in knowledge of the actors, in addition to a lack of infrastructure and communication device. Since the historical drama of Thalidomide, international scientific community immediately realized that despite clinical trials, with a good follow up, a drug remains dangerous after being placed on the market. Therefore, in order to avoid harm to patients and improve public health, it is essential to establish a well-organized system of pharmacovigilance. It also appears as an essential component of an effective drug regulation system, clinical practice and health programs. Despite the call from WHO to strengthen national pharmacovigilance systems, developing countries are still struggling to seriously include ir pharmacovigilance in their health information system. This goes through the assessment of health professional knowledge and practice of pharmacovigilance. Cameroon, this is not known, thus our motivation to carry out this study aiming to assess the health professional knowledge and practice of pharmacovigilance in this country.

## Methods

From June to September 2013, we carried out a study on a sample drawn from a synthetized database of 12526 health professionals composed of physicians, pharmacists and dentists in registered as shown in the national health statistics report of Cameroon in 2010. We asserted that 10% of prescribers have no “knowledge” of pharmacovigilance. We therefore calculated a sample of 138 patients, with an error margin of 5% and a confident level of 95% using Epi Info statcalc. We extended our selection to the four groups as not only physicians prescribe drugs in Cameroon. We sent out to the sample population 200 questionnaires and received 149 exploitable responses. We analyzed data using IBM SPSS 20.0 software. We applied a 0.05 threshold for p-value for statistical significance when comparing proportions.

## Results

**Figure 1:**
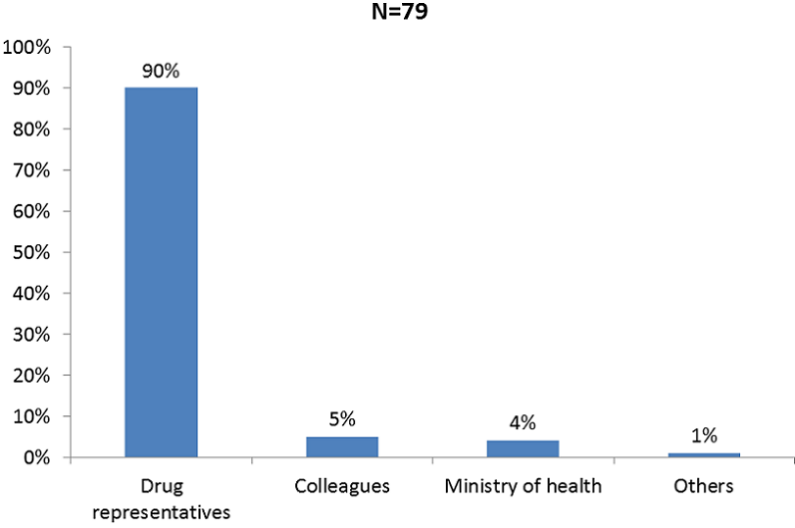
Place of side effects cases declaration

Our sample was composed of 55% of males. We had 57% of physicians, 23% of pharmacists, 12% of nurses and 8% of dentists. Nearly all our respondents had already prescribed, as well as observed a side effect, which in half of cases was from a drug prescribed by them (Table 1). Of the reason the cases were not declared, not knowing to whom to declare was the first, followed by lack of adequate contact of where to declare (Table 2). Nearly all the declared cases were done through the drug representatives of pharmaceutical companies (Figure 1). Physicians and pharmacists gave the less wrong definition of pharmacovigilance than dentists and nurses. More of physicians and pharmacists also believed there was no functioning pharmacovigilance system in Cameroon (Table 3).

**Table 1:** Prescription behavior (“yes answers”)

	n	Proportion
Have you ever prescribed or administered a drug?	145	97,9%
Have you ever observed a side effect?	142	96,5%
Have you ever declared a side effect?	148	62,2%
Were you the one who prescribed the causing drug?	129	50,4%

Nearly all our respondents had already prescribed, as well as observed a side effect, which in half of cases was from a drug prescribed by them

**Table 2:** Reason of non-declaration of side effects

	n	Proportion
Did not know to whom to declare	55	18,2%
Did not have the adequate contact to declare	56	11,0%
Did not foresee the importance	55	09,0%
Did not know it should be declared	54	05,5%
Did not have the « means » to declare	56	03,6%
The patient did not want the side effect to be declared.	55	05,5%

Of the reason the cases were not declared, “not knowing to whom to declare” was the first, followed by “lack of adequate contact of where to declare”

**Table 3:** Prescribers Status of knowledge on Pharmacovigilance

	Physicians	Pharmacists	Nurses	Dentists
n	85	34	18	12
Have you hear of pharmacovigilance? (Answers: “yes”)	95,3%	96,1%	55,5%	83,3%
Could you define pharmacovigilance? (Wrong definition)	15,2%	26,5%	61,1%	58,3%
Does a pharmacovigilance systems exists in Cameroon (Answers: “no”)	54,1%	17,6%	44,4%	58,3%
Is the Cameroon Pharmacovigilance system functional? (Answer: “No”)	78,8%	97,0%	38,9%	25,0%

Physicians and pharmacists gave the less wrong definition of pharmacovigilance than dentists and nurses. More of physicians and pharmacists also believed there was no functioning pharmacovigilance system in Cameroon

## Discussion

This study which is the first in Cameroon highlighted a certain number of important facts. Of the entire sample, only few had never heard about pharmacovigilance, despite nearly all of them have ever prescribed a drug or even observed side effects. It shows the interest to build on the level of awareness of health workers on the matter. Though, having about a tenth of the sample not being aware of it still also shows that the concept may not be well though in our faculties or once back home after training, our prescribers do not apply the concept in their practice. Such an observation was made in a study in Nigeria on a survey in prescribers [10]. It shows the interest of training continuously our prescribers on the topic. In this study, pharmacists appear to be the most informed about pharmacovigilance. This may be due to the fact that from their basic training, they are the most implicated in drug, and later on, in terms of contact, delivery, preparation and explaining to the patients. An important reason for non-declaring side effects was lack of adequate contact or information on where to declare. The entire sample believes it is important to declare side effects, but most of them declared to drug representatives from pharmaceutical companies. These representatives belong to companies with internal developed pharmacovigilance structures, required at national level through registration conditions. Building on such an operational scheme could be a possible channel to develop a good pharmacovigilance system while waiting for implementation of well-functioning one at national level. Half of the respondent in our study were not aware of an existing functional pharmacovigilance system in Cameroon; which is less than findings in a survey in a similar poor resource setting in 2013 [11]. Informing pharmaceutical companies’ drug representatives rather than national system shows on one hand the willing to declare, but also the lack of strict regulation on procedures in reporting such events. The only few reported cases received at ministry of health levels come from vertical programs in general, as observed in vaccine program side effects follow-up [12]. Comparably to a study in Senegal in 2013 [13] most of the dentists in our country are not aware of such a system though they are potential important prescribers as shown in similar settings.

## Conclusion

Pharmacovigilance is a public health problem in Cameroon and the problem due to lack of good knowledge and practice of prescribers precisely physicians, pharmacists, nurses, and dentists who are not always aware of an existing pharmacovigilance system in Cameroon.
